# *Angelica sinensis* is effective in treating diffuse interstitial pulmonary fibrosis in rats

**DOI:** 10.1080/13102818.2014.957487

**Published:** 2014-10-29

**Authors:** Lu Wang, Yanmei Sun, Cailian Ruan, Bofeng Liu, Lin Zhao, Xiujuan Gu

**Affiliations:** ^a^ Department of Human Anatomy, School of Medical Sciences, Yan'an University, Yan'an, Shaanxi, P.R. China; ^b^ Department of Internal Medicine, Qinghua Hospital, Xi'an, Shaanxi, P.R. China; ^c^ Department of Inspection Division, Yan'an University Affiliated Hospital, Yan'an, Shaanxi, P.R. China

**Keywords:** diffuse interstitial pulmonary fibrosis, *Angelica sinensis*, nuclear factor

## Abstract

The aim of this study was to investigate the therapeutic effect of *Angelica sinensis* on a rat model of diffuse interstitial pulmonary fibrosis induced by bleomycin A5. The mechanism by which *A. sinensis* exerts its effect is also discussed. A diffuse interstitial pulmonary fibrosis model was established in 36 male Wistar rats by an endotracheal injection of bleomycin A5 (5 mg/kg). Then, these rats were randomly divided into the model group (*n* = 18) and the treatment group (treated with *A. sinensis* after modelling, *n* = 18). Control rats (*n* = 6) received an equal volume of saline. Hematoxylin–eosin (HE) staining was performed to analyse alveolitis and Masson staining, to observe pulmonary fibrosis. Collagen content was determined by hydroxyproline assay. Nuclear factor kappa B (NF-κB) activity was measured by electrophoretic mobility shift assay. Transforming growth factor-β (TGF-β) expression at mRNA level was detected by northern blotting and at protein level by enzyme-linked immunosorbent assay. The results obtained showed that the alveolitis and pulmonary fibrosis of the rats treated with *A. sinensis* was significantly alleviated compared with that of the rats in the model group. Treatment with *A. sinensis* also lowered the content of collagen, decreased NF-κB activity in alveolar macrophages and reduced the TGF-β expression at the mRNA and protein level. These results indicated that *A. sinensis* is effective in treating and alleviating interstitial pulmonary fibrosis, possibly by lowering collagen, inhibiting the activity of NF-κB and reducing the TGF-β expression.

## Introduction

Diffuse interstitial pulmonary fibrosis is a kind of inflammatory disease that is caused by many factors, leading to pathological changes in interstitial tissues, alveolar epithelial cells or pulmonary veins. Its causes include the inhalation of inorganic dust, such as asbestos and coal, organic dust, such as triuridaceae pollen and cotton dust and gases, such as smoke and sulphur dioxide; infections by viruses, bacteria, fungi, and parasites; as well as drug influence and radioactive damage. Diffuse interstitial pulmonary fibrosis starts from alveolitis. After the activation of macrophages in pulmonary alveolus by stimulations, they secrete multiple cytokines, such as transforming growth factor-β (TGF-β), induce inflammatory responses and stimulate the hyperplasia of fibroblasts, leading to the secretion of fibronectin and the formation of fibrosis. Nuclear factor kappa B (NF-κB), a kind of DNA-binding protein, is an important regulatory factor for gene transcription in eukaryotic cells. Recent studies indicated that NF-κB participated in regulating immune responses and inflammation, by promoting the transcription of inflammatory genes and the TGF-β gene,[1] which played important roles in alveolitis. In recent years, it was reported that *Angelica sinensis* was effective in treating diffuse interstitial pulmonary fibrosis, possibly by preventing or reversing the fibrosis of the tissues.[2] In this study, we observed the therapeutic effect of *A. sinensis* in rat models with diffuse interstitial pulmonary fibrosis and investigated possible mechanisms by which *A. sinensis* exerted its effect.

## Materials and methods

### Animals

Clean-grade healthy male Wistar rats, with body weights between 200 and 250 g, were provided by the Center for Laboratory Animals, Peking University Health Science Center. All animal experiments were conducted according to the ethical guidelines of Yan'an University.

### Drugs

Bleomycin A5, 8 mg/vial, was produced by Tianjin Taihe Pharmaceutical Co., Ltd. (Tianjin, China). Liquid injection of *A. sinensis* was produced by Shanxi Yongji Pharmaceutical Company (Yongji, Shanxi, China).

### Reagents and instruments

NF-κB probes were obtained from Promega (USA). Enzyme-linked immunosorbent assay (ELISA) kit for TGF-β was provided by Jingmei Company (Shenzhen, China). DYY-3A electrophoresis chamber was purchased from Beijing Liuyi Instrument Factory (Beijing, China). Constant voltage and current DF-D electrophoresis apparatus was purchased from Beijing Dongfang Instrument Factory (Beijing, China). Model 511 of ELISA analyser was purchased from Third Analytical Instrument Factory of Shanghai (Shanghai, China).

### Animal model establishment and sample preparation

Thirty-six male Wistar rats were etherised, subjected to endotracheal intubation, endotracheally injected with bleomycin A5 (5 mg/kg) and used as models of diffuse interstitial pulmonary fibrosis. These rats were randomly divided into two even groups. For the model group, 18 rats were sacrificed at the end of weeks 1, 2 and 4. For the treatment group, 18 rats were injected with *A. sinensis* via a nasogastric tube at 100 mg/kg body weight and at 2 h after the establishment of the model. Then, the same doses were injected once a day before the rats were sacrificed at the end of weeks 1, 2 and 4. A control group of six rats underwent endotracheal intubation for the injection of 0.5 mL saline and were sacrificed after one week.

After sacrifice, the lung tissues were excised for pathological observation, determination of collagen and northern blotting hybridisation. Bronchoalveolar lavage fluid (5 mL) was collected and used for ELISA assay. Pulmonary alveolar macrophages (about 5 × 10^6^ to 1 × 10^7^) were used for the NF-κB activity determination.

### Histopathological evaluation

The right upper lobe of lung tissues was obtained from rats for alveolitis and pulmonary fibrosis assay. Briefly, lung tissues were fixed, embedded in paraffin and cut into tissue sections. For histology, the sections were stained with hematoxylin–eosin (HE) and Masson's trichrome. The severity of alveolitis and pulmonary fibrosis was assessed according to the scoring method described by Szapiel et al. [3], with some modifications.

The degree of alveolitis and pulmonary fibrosis were determined for semi-quantitative pathological analysis, as previously described in [4]. The grading criteria used for alveolitis were: Degree 1, no alveolitis; Degree 2, mild alveolitis, affecting <20% of the total lung, with the infiltration of mononuclear cells into the widened alveolar septa and limited to localised regions with the involvement of nearby pleural areas; Degree 3, moderate alveolitis, affecting an area of 20%–50%, with greater pleural involvement; Degree 4, severe alveolitis, involving an area of >50%, with occasional monocytes in the alveolar space and bleeding caused by consolidation. The scoring criteria used for fibrosis were: Degree 1, no fibrosis; Degree 2, mild fibrosis, affecting an area of <20% of the whole lung, with fibrosis involving the pleura and subpleural interstitium and the disorders of alveolar structure; Degree 3, moderate fibrosis, involving an area of 20%–50%, with localised areas of fibrosis extending from the pleura; Degree 4, severe fibrosis, involving an area of >50%, with the fusion of alveolar spaces.

### Determination of the collagen content

Hydroxyproline content in pulmonary tissues was used to quantify the lung collagen content and was measured colorimetrically by a previously described method, with some modifications.[5–7] Briefly, lung parenchyma samples from mice were weighed and hydrolysed at 110 °C for 24 h to release hydroxyproline from collagen. Then, samples were mixed with citrate–acetate buffer (5% citric acid, 1.2% glacial acetic acid, 7.25% sodium acetate and 3.4% sodium hydroxide) and chloramine-T solution (1.4% chloramine-T, 10% N-propanol and 80% citrate–acetate buffer). The mixture was incubated for 5 min at room temperature. After incubation, 4-(dimethylamino)benzaldehyde was added to react with hydroxyproline chromogen. Absorbance was measured at 560 nm. Standard curves were generated for each experiment, using reagent hydroxyproline as a standard and the hydroxyproline contents of samples were calculated according to the standards. The content of collagen in pulmonary tissues was then expressed as micrograms of hydroxyproline per gram of wet lung weight (mg/g).

### Electrophoretic mobility shift assay (EMSA)

For the preparation of nuclear extracts, alveolar macrophage cells were lysed and incubated in hypotonic low salt buffer and then high salt buffer. The nuclear extracts were separated by centrifugation. For electrophoretic mobility shift assay (EMSA), nuclear extracts were incubated with radioactive labelled NF-κB oligonucleotide probes. After binding, the samples were separated by non-denaturing polyacrylamide gel electrophoresis. According to the method described in [8], the gel was analysed using an IBAS-2000 image analysis system.

### Northern blotting

The expression of mRNA was detected by northern blotting.[9] Briefly, total RNA was extracted from pulmonary tissues using Trizol reagent. Then, RNA was denatured and separated by electrophoresis, using a 1% agarose gel containing 1.9% formaldehyde. The RNA was transferred to a nylon membrane by capillary blotting and was cross-linked by ultraviolet radiation. Equal gel loading and the integrity were verified by ethidium bromide staining of the 18S and 28S RNAs. Hybridisations were performed in a rotating drum in a temperature-controlled oven. Blots were washed twice at room temperature in 2× saline sodium citrate (SSC) buffer (1× SSC: 0.15 mol/L of NaCl, 15 mmol/L of sodium citrate) containing 0.1% sodium dodecyl sulfate (SDS), once in 1× SSC buffer containing 0.1% SDS at 68 °C and once in 0.1× SSC containing 0.1% SDS at 60 °C. Membranes were exposed to films for 24 to 48 h and analysed using National Institutes of Health (NIH) Imaging Software.

### ELISA

The assay was carried out according to the manual provided by the ELISA kit. Briefly, bronchoalveolar lavage fluid samples were added to the pre-coated microplate and incubated at 4 °C overnight. After washing five times, detection antibody was added to the wells. After incubation at room temperature for 1 h, the microplate was washed again. Then, horseradish peroxidase-conjugated antibody was added and incubated at room temperature for 30 min. After washing five times, substrate solution was added and incubated at room temperature for 15 min. Finally, stop solution was added to stop colour development and the plate was read at 450 nm with a Model 511 ELISA analyser. The standard curve was generated by twofold serial dilutions of the standard samples. The content of TGF-β was calculated according to the standard curve.

### Statistical analysis

The counting and measurement data were expressed as means ± standard deviation (SD). *Chi*-square test and *t*-test were performed using Statistical Package for the Social Sciences (SPSS 13.0) software. A *P* value less than 0.05 was considered statistically significant.

## Results and discussion

### A. *sinensis* treatment alleviates pathohistological changes

To investigate the degrees of pathohistological changes in alveolitis and interstitial pulmonary fibrosis, HE staining and Masson staining were performed. The grading results are shown in Table 1. The pulmonary alveolus of the control group was normal, without pathological changes, such as alveolitis and interstitial pulmonary fibrosis. The alveolar cavity and pulmonary interstitial tissues of the model group after one week showed inflammatory cell infiltration and the epithelial cells fell off the pulmonary alveolus, exhibiting severe alveolitis. At week 4 after modelling, there were fibroblast aggregation, increased collagen precipitation, alveolar structural damage and obvious fibrosis in the model group. The pathological change of the model group after two weeks was between those after one and four weeks. The treatment group showed alleviated pathological changes and the decreased pathological area, compared with the model group at the same time period. The main pathological change of the model group in week 1 was alveolitis, without obvious fibrosis.

**Table 1.** Degrees of alveolitis and interstitial pulmonary fibrosis analysed by HE and Masson staining.

			Alveolitis classification				Pulmonary fibrosis classification			
Groups		*N*	Degree 0	Degree 1	Degree 2	Degree 3	Degree 0	Degree 1	Degree 2	Degree 3
Control	Weeks 1, 2, 4	6	6	0	0	0	6	0	0	0
Model	Week 1	6	0	0	1	5	3	2	1	0
	Week 2	6	0	0	2	4	1	3	1	1
	Week 4	6	0	2	3	1	0	0	3	3
Treatment	Week 1	6	0	2	3	1	4	1	1	0
	Week 2	6	0	2	3	1	3	2	1	0
	Week 4	6	0	4	2	0	0	2	2	2

### Treatment with *A. sinensis* lowers the collagen content

To test the effect of *A. sinensis* on the content of collagen in the lung tissues, the content of hydroxyproline was measured. Then, the content of collagen in pulmonary tissues was expressed as the micrograms of hydroxyproline per gram of wet lung weight (mg/g) (Table 2). The content of collagen in the model group at the end of week 4 was increased significantly compared with the control group (*P* < 0.05). The content of collagen in the treatment group at the end of week 4 was significantly reduced compared with that in the model group (*P* < 0.01). These data indicate that treatment with *A. sinensis* lowered the collagen content.

**Table 2.** Content of collagen in the lung tissues (mg/g).

Groups	Control	Model	Treatment
Week 1	94.61 ± 6.52	101.71 ± 8.09	98.73 ± 9.95
Week 2	93.65 ± 5.57	114.83 ± 5.47	102.52 ± 4.26
Week 4	94.72 ± 5.31	167.32 ± 6.44*	96.34 ± 11.17^#^

*P < 0.05, compared with the control group.

^#^ *P* < 0.01, compared with the model group.

### Treatment with *A. sinensis* decreases the NF-κB activity in alveolar macrophages

To determine how *A. sinensis* affects the NF-κB activity, EMSA was performed. Quantitative results are shown in Table 3. The NF-κB activity in alveolar macrophages of the model group was increased significantly compared with that in the control group after one week (*P* < 0.05). It was reduced to normal after two weeks and decreased to lower than normal after four weeks. And there was no significant difference in the NF-κB activity at weeks 2 and 4 between the control group and the model group. The NF-κB activity in alveolar macrophages of the treatment group was significantly lower than that of the model group after one week (*P* < 0.05). These data suggest that *A. sinensis* treatment decreased the NF-κB activity in alveolar macrophages.

**Table 3.** NF-κB activity in alveolar macrophages.

Groups	Control	Model	Treatment
Week 1	4.56 ± 2.25	32.83 ± 5.48*	18.74 ± 5.41^#^
Week 2	4.81 ± 1.85	6.73 ± 3.24	3.36 ± 0.93
Week 4	5.23 ± 1.37	3.46 ± 4.22	4.27 ± 1.23

*P < 0.05, compared with the control group.

^#^ *P* < 0.05, compared with the model group.

### Treatment with *A. sinensis* reduces the expression of TGF-β mRNA in lung tissues

**Figure 1.**
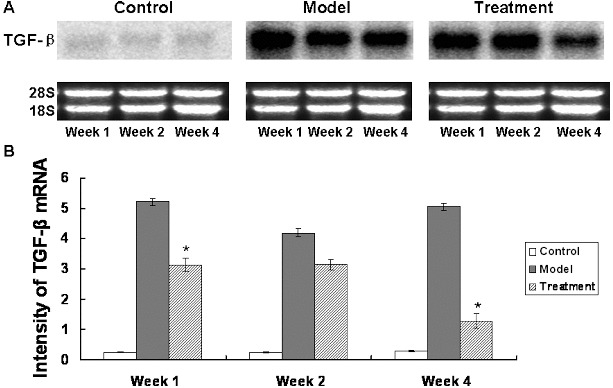
Northern blot analysis of the TGF-β expression at the mRNA level. Pulmonary tissues of the control group, the model group and the treatment group were collected at week 1, week 2 and week 4 after modelling. Total RNAs were extracted from these pulmonary tissues for northern blotting analysis. (A) Representative results of northern blots probed with TGF-β; ethidium bromide staining of 18S and 28S rRNA indicated the integrity and equal gel loading of RNA. (B) Quantitative northern blots results of TGF-β intensity. Compared with the model group, *P < 0.05.

To study how *A. sinensis* affects the expression of TGF-β mRNA, northern blotting assay was conducted. Equal gel loading and the integrity were verified by ethidium bromide staining of the 18S and 28S RNAs. Representative northern blotting results are shown in Figure 1(A) and quantitative northern blotting results are shown in Figure 1(B). Northern hybridisation results suggested that normal lung tissues basically did not express TGF-β. The expression of TGF-β mRNA was significantly increased in the model group within one or two weeks, with the peak value appearing after week 1. The expression of TGF-β mRNA in the treatment group was significantly lower than that in the model group at weeks 1 and 4 (*P* < 0.05) (Figure 1). These results demonstrate that treatment with *A. sinensis* reduced the expression of TGF-β mRNA in lung tissues.

### Treatment with *A. sinensis* lowers the level of TGF-β protein in bronchoalveolar lavage fluid

To define the effects of *A. sinensis* on the TGF-β protein production, ELISA assay was performed in bronchoalveolar lavage fluid. The level of TGF-β protein in bronchoalveolar lavage fluid in the model group at weeks 1 and 2 was significantly enhanced compared with that of the control group (*P* < 0.05). Its peak value appeared after one week. The level of TGF-β protein in bronchoalveolar lavage fluid in the treatment group at weeks 1 and 2 was significantly lowered compared with that of the model group at the same period (*P* < 0.05) (Table 4). These data show that treatment with *A. sinensis* lowered the level of TGF-β protein in bronchoalveolar lavage fluid.

**Table 4.** TGF-β protein level in bronchoalveolar lavage (μg/L).

Groups	Control	Model	Treatment
Week 1	89.23 ± 32.4	384.22 ± 78.2*	123.92 ± 34.14^#^
Week 2	92.87 ± 30.1	291.25 ± 89.27*	112.86 ± 21.28^#^
Week 4	90.34 ± 28.8	102.30 ± 23.21	119.63 ± 34.36

*P < 0.05, compared with the control group.

^#^ *P* < 0.05, compared with the model group.

### Comparative analysis

*A. sinensis* is a kind of perennial herb used in Chinese medicine as an important blood replenishment and circulation drug. It is widely recognised for its effects in resisting platelet aggregation, promoting the solution of plasma fibrinogen, increasing blood volume, improving microcirculation and regulating the immune system.[10,11] The active ingredient of *A. sinensis*, ferulic acid, has a significant effect in anti-lipid peroxidation.[12] It has also been reported that *A. sinensis* is effective in treating interstitial pulmonary fibrosis. However, the underlying mechanism is not fully understood. In this study, we investigated the role and the underlying mechanism of *A. sinensis* treatment on interstitial pulmonary fibrosis induced by bleomycin A5.

Our results that alveolitis and pulmonary fibrosis was significantly alleviated by *A. sinensis* and that collagen content was also decreased after *A. sinensis treatment* are consistent with previous studies [2,13] and further confirm the anti-fibrosis effect of *A. sinensis*. Through genome-wide transcriptional profiling, Cabrera et al. [14] found 533 significantly changed genes involved in the progression and resolution of bleomycin-induced lung fibrosis, including genes mediating inflammation and fibrosis. NF-κB is an important inflammatory mediator and activated in bleomycin induced fibrosis.[15] In our study, the NF-κB activity in alveolar macrophages in the model of pulmonary fibrosis induced by bleomycin A5 was significantly elevated compared with that of the control group at week 1. Meanwhile, compared with the model group, NF-κB activity in the treatment group was significantly inhibited. TGF-β plays a key role in experimental animal models of pulmonary fibrosis [16,17] and is regarded as a key pro-fibrotic mediator.[18] Interestingly, we found that the TGF-β expression at the mRNA and protein level was both significantly increased in the model group. However, after treatment with *A. sinensis*, the increase in the TGF-β expression was reduced. Similarly, Chitra et al. [19] reported the beneficial effects of berberine against fibrosis through activating Nrf2 and suppressing NF-κB-dependent TGF-β activation. These results indicate the role of NF-κB and TGF-β in bleomycin-induced fibrosis. Taken together, these observations demonstrate that *A. sinensis* treatment suppressed the activation of NF-κB and the expression of TGF-β. This may prove to be one of the underlying mechanisms of the anti-fibrosis effect of *A. sinensis*. However, the downstream mediators of NF-κB and TGF-β are not studied here and still need further investigation.

## Conclusions

This study reported that *A. sinensis* could alleviate alveolitis and pulmonary fibrosis induced by bleomycin. These beneficial effects of *A. sinensis* were mediated by inhibiting NF-κB-dependent inflammatory and TGF-β1-mediated fibrotic events. Our data provide experimental evidence for the further investigation of the anti-fibrosis effect of *A. sinensis*.
